# Pre-existing antibody-mediated adverse effects prevent the clinical development of a bacterial anti-inflammatory protein

**DOI:** 10.1242/dmm.045534

**Published:** 2020-09-28

**Authors:** Angelino T. Tromp, Yuxi Zhao, Ilse Jongerius, Erik C. J. M. Heezius, Pauline Abrial, Maartje Ruyken, Jos A. G. van Strijp, Carla J. C. de Haas, András N. Spaan, Kok P. M. van Kessel, Thomas Henry, Pieter-Jan A. Haas

**Affiliations:** 1 Department of Medical Microbiology, University Medical Center Utrecht, 3584CX Utrecht, The Netherlands; 2 Sanquin Research, Department of Immunopathology, 1006AD Amsterdam, The Netherlands; 3 Landsteiner Laboratory, Amsterdam UMC, University of Amsterdam, 1105AZ Amsterdam, The Netherlands; 4 Centre International de Recherche en Infectiologie, Inserm, U1111, Université Claude Bernard Lyon 1, CNRS UMR5308, Ecole Normale Supérieure de Lyon, Université Lyon, 69007 Lyon, France; 5 St. Giles Laboratory of Human Genetics of Infectious Diseases, Rockefeller Branch, The Rockefeller University, New York, NY 10065, USA

**Keywords:** C5aR chemotaxis, CHIPS, Clinical trials, Humanized mouse, Immune complex

## Abstract

Bacterial pathogens have evolved to secrete strong anti-inflammatory proteins that target the immune system. It was long speculated whether these virulence factors could serve as therapeutics in diseases in which abnormal immune activation plays a role. We adopted the secreted chemotaxis inhibitory protein of *Staphylococcus aureus* (CHIPS) as a model virulence factor-based therapeutic agent for diseases in which C5AR1 stimulation plays an important role. We show that the administration of CHIPS in human C5AR1 knock-in mice successfully dampens C5a-mediated neutrophil migration during immune complex-initiated inflammation. Subsequent CHIPS toxicology studies in animal models were promising. However, during a small phase I trial, healthy human volunteers showed adverse effects directly after CHIPS administration. Subjects showed clinical signs of anaphylaxis with mild leukocytopenia and increased C-reactive protein concentrations, which are possibly related to the presence of relatively high circulating anti-CHIPS antibodies and suggest an inflammatory response. Even though our data in mice show CHIPS as a potential anti-inflammatory agent, safety issues in human subjects temper the use of CHIPS in its current form as a therapeutic candidate. The use of staphylococcal proteins, or other bacterial proteins, as therapeutics or immune-modulators in humans is severely hampered by pre-existing circulating antibodies.

## Abstract (precis)

**Summary:** Chemotaxis inhibitory protein of *Staphylococcus aureus* dampens C5a-mediated responses in human-C5aR1 knock-in mice. Feasibility studies in a human phase I trial failed due to high levels of existing natural antibodies causing adverse effects.

## INTRODUCTION

The human immune system is a well-balanced and effective network of cells, tissues and organs, and plays a crucial role in the continuous fight against invading microbes (Chaplin, 2010). On the other hand, the survival of microbial pathogens depends on their ability to withstand attacks by the immune system (Hornef et al., 2002; Rooijakkers and van Strijp, 2007). Successful pathogenic bacteria have co-evolved with the host and acquired complex methods of subverting and suppressing the immune system (Rooijakkers and van Strijp, 2007). The deployment of strong and specific immune-modulatory proteins by bacteria have shown to be effective immune suppressors *in vitro* and *in vivo* in mice (Rooijakkers and van Strijp, 2007; Laarman et al., 2010; Kobayashi et al., 2018; Veldkamp and van Strijp, 2009). Considering that abnormal or excessive activation of the immune system can lead to inflammatory diseases, it was long speculated whether these bacterial virulence factors could serve as anti-inflammatory therapeutics in conditions in which undesirable immune activation plays a role (Laarman et al., 2010). Over the years, studies have alluded to the therapeutic potential of various bacterial proteins that normally play a role in immune evasion (Laarman et al., 2010). However, as bacterial-derived proteins will induce antibody responses, it remains enigmatic whether these proteins can indeed serve as a means for anti-inflammatory treatments in humans. Examples of known pathogenic bacteria that secrete immune-evasion proteins are *Streptococcus pneumoniae*, *Pseudomonas aeruginosa*, *Neisseria gonorrhoeae* and *Listeria monocytogenes* (Kobayashi et al., 2018; von Pawel-Rammingen et al., 2002; Bardoel et al., 2012; Carrero et al., 2004). However, secreting more than 35 immune-evasion molecules, *Staphylococcus aureus* is the text-book example of immune evasion by bacteria (Koymans et al., 2016).

*Staphylococcus aureus*, a common colonizer of human skin and the human nose, as well as a human pathogen, has evolved to secrete an arsenal of virulence factors that target the human immune system (Spaan et al., 2013a). One extensively described and well-studied *S. aureus* virulence factor is the chemotaxis inhibitory protein of *Staphylococcus aureus* (CHIPS). CHIPS binds to the N-terminus of human C5AR1 with high affinity (KDa=1.1 nM) and functionally blocks the interaction with C5a, thus preventing C5AR1 stimulation and antagonizing chemotaxis (de Haas et al., 2004; Postma et al., 2005, 2004). Besides playing a role in chemotaxis as a response to microbial invasion, C5AR1 is involved in a variety of other inflammatory processes. Upregulation of C5AR1 in internal organs during the onset of sepsis, together with the excessive release of C5a, was proposed to lead to multi-organ failure and death in rats (Riedemann et al., 2002; Guo et al., 2003). The blockade of C5AR1 with polyclonal anti-C5AR1 antibodies was protective and increased survival in an animal sepsis model (Riedemann et al., 2002). C5a and C5AR1 have also been associated with disease processes such as ischemia-reperfusion injury, rheumatoid arthritis, asthma, immune complex diseases, neurodegeneration and Alzheimer's disease (Klos et al., 2009; Guo and Ward, 2005; Farkas et al., 2003; Huber-Lang et al., 2001a; Woodruff et al., 2008). Targeting of C5AR1 has been shown to be beneficial in some of these disease processes in animals, emphasizing the relevance of C5AR1 as a therapeutic target (Guo et al., 2004; Huber-Lang et al., 2001b; Fonseca et al., 2009; Klos et al., 2013).

The properties of CHIPS to inhibit human C5AR1 with high specificity and affinity makes it an example of a promising anti-inflammatory drug candidate for diseases in which C5AR1 stimulation plays an important role. Previous studies have shown that the antagonistic activity of CHIPS on mouse C5ar1 is 30-fold lower compared to human C5AR1-expressing cells (de Haas et al., 2004). This human specificity of CHIPS has hampered the assessment of CHIPS *in vivo* during inflammation and infection. Here, we report the application of a transgenic human C5AR1 knock-in mouse (hC5aR1^KI^) to assess CHIPS as a model anti-inflammatory compound in C5AR1-mediated diseases. Furthermore, we investigate the safety and efficacy of CHIPS in a phase I, randomized double-blind placebo-controlled study in humans.

## RESULTS

### CHIPS binds hC5aR1^KI^ murine neutrophils and inhibits stimulation by murine C5a

**Fig. 1.**
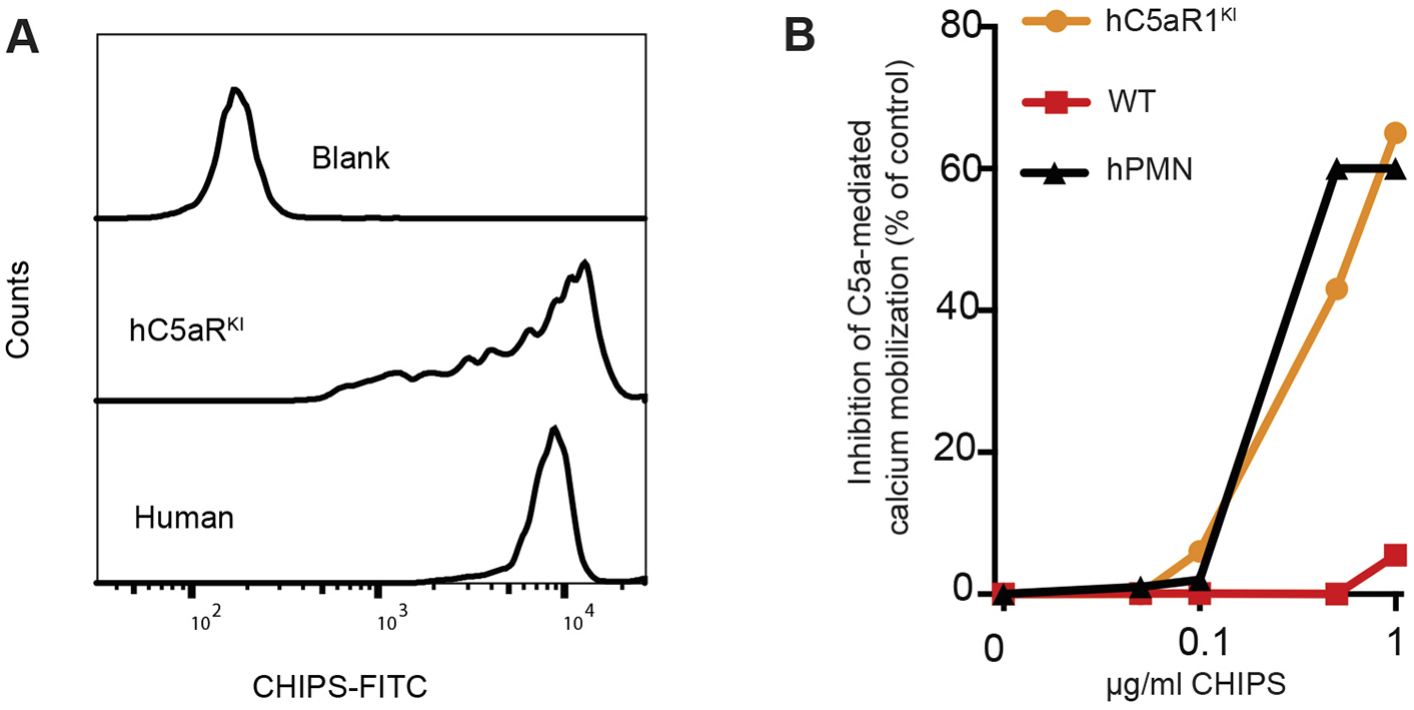
**CHIPS binds and inhibits hC5aR1^KI^ murine neutrophils at levels comparable to those with human neutrophils.** Quantification of hC5aR1 expression in hC5aR1^KI^ mice showed similar expression levels compared to human leukocytes (Tromp et al., 2018). Furthermore, hC5aR1^KI^ murine neutrophils responded normally to both murine C5a (mC5a) and human C5a as measured by Ca mobilization (Tromp et al., 2018). (A) hC5aR1^KI^ bone marrow neutrophils and human blood neutrophils were isolated and incubated with 3 µg/ml histidine-tagged CHIPS followed by anti-histidine-fluorescein isothiocyanite (FITC) antibodies. Cells were analyzed by flow cytometry and the FITC fluorescent signal was depicted as histograms. (B) As our hC5aR1^KI^ murine model generates mC5a, the assessment of CHIPS inhibition was performed by mC5a stimulation. Bone marrow neutrophils of hC5aR1^KI^, wild-type (WT) mice and human neutrophils were pre-incubated with CHIPS at the indicated concentration and subsequently stimulated with murine C5a (10-8M). The basal fluorescence level was first measured for each sample before the addition of murine C5a. The C5a-mediated calcium influx was analyzed by flow cytometry using Fluo-4AM. The average Fluo-4AM fluorescent signal was used to calculate CHIPS-mediated inhibition of C5a responses. One experiment representative of two independent experiments is shown.

In order to validate the suitability of our hC5aR1^KI^ mouse (Tromp et al., 2018) as a model to evaluate CHIPS *in vivo*, we first assessed the activity of CHIPS on hC5aR1^KI^ murine neutrophils. To this end, the binding of CHIPS to bone marrow-derived hC5aR1^KI^ murine neutrophils was determined and compared with human neutrophils isolated from peripheral blood. We confirmed that CHIPS binds to hC5aR1^KI^ murine neutrophils at levels comparable to those observed with human neutrophils (Fig. 1A). To further assess the activity of CHIPS, the inhibition of hC5aR1 was determined in human and hC5aR1^KI^ murine neutrophils. Wild-type murine neutrophils respond normally to mC5a but CHIPS is ineffective in inhibiting mC5a-mediated Ca mobilization of these mC5aR-expressing cells (Fig. 1B). Correspondingly, CHIPS inhibition of mC5a-mediated Ca mobilization of hC5aR1^KI^ neutrophils reflected that observed with human neutrophils (Fig. 1B). Hereby, we confirm the binding and inhibition of hC5aR1^KI^ murine neutrophils by CHIPS, proving that our hC5aR1^KI^ I mouse is a suitable model to assess CHIPS activity *in vivo*.

### CHIPS inhibits C5aR mediated neutrophil migration *in vivo*

**Fig. 2.**
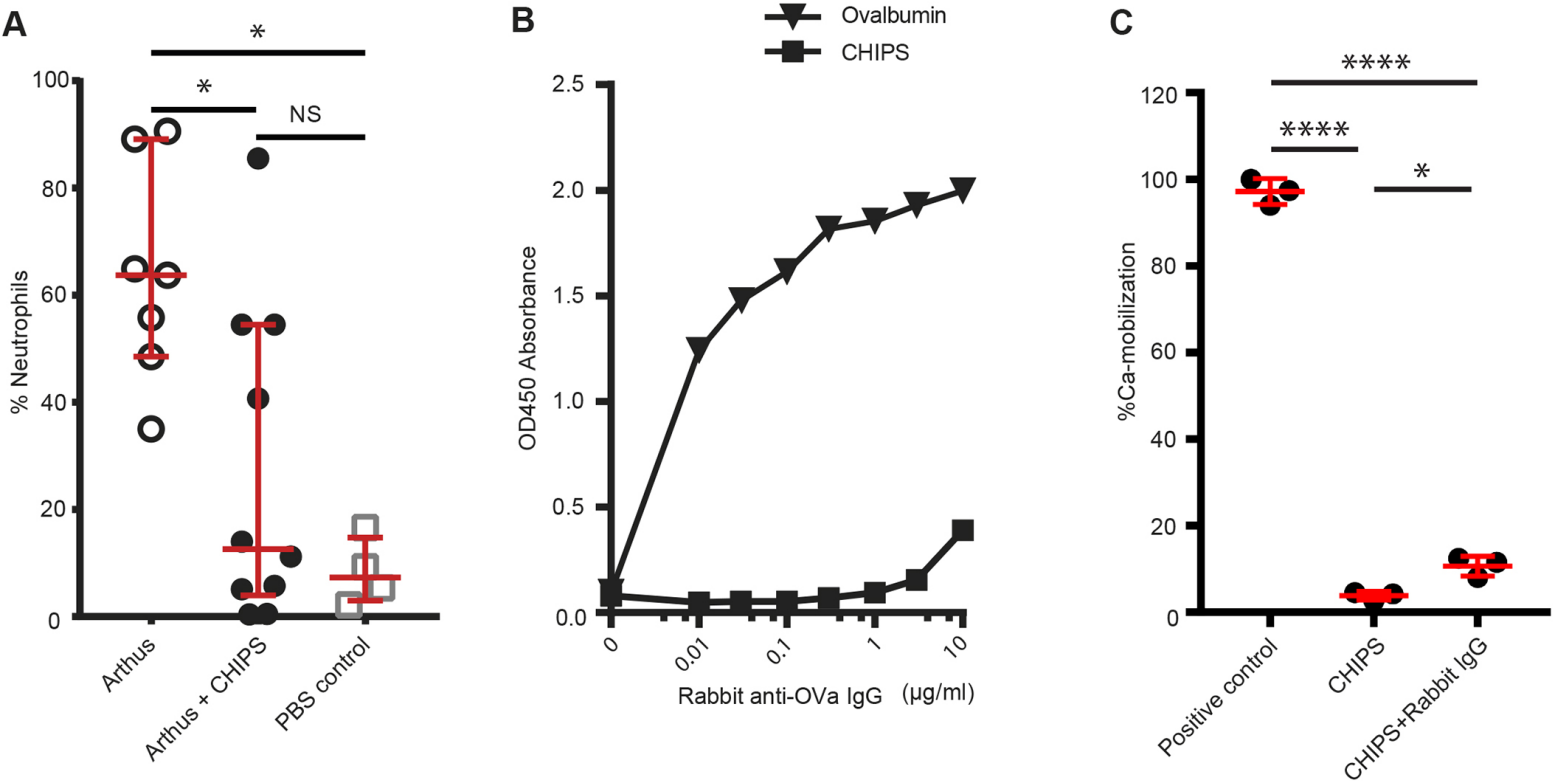
**CHIPS inhibits neutrophil migration *in vivo*.** (A) CHIPS (60 μg, *n*=10) was injected i.p., together with OVA i.v. in hC5aR1^KI^ mice 30 min before inducing the Arthus reaction. Samples were compared to mice that did not receive CHIPS (*n*=7). Control mice (*n*=4) received PBS i.v. and i.p. Peritoneal cavity lavage was performed 6 h post Arthus induction. The percentage of neutrophil influx was analyzed by flow cytometry by gating on a CD45^+^GR-1^+^F4/80^−^ population, and depicted as a percentage of total leukocytes (CD45^+^) retrieved after peritoneal lavage. All groups consisted of equal numbers of female and male mice. The median with interquartile range of the combined data from two independent experiments is shown. (B) The presence of anti-OVA and anti-CHIPS antibodies in the rabbit anti-OVA IgG fraction was determined by ELISA. (C) To detect neutralizing anti-CHIPS antibodies in the rabbit anti-OVA IgG, CHIPS (500 ng/ml) was incubated with 10 µg/ml rabbit anti-OVA IgG or PBS. Subsequently, Fluo-4AM-labeled human neutrophils were incubated with CHIPS/Rabbit IgG or CHIPS/PBS and challenged with human C5a. Ca mobilization was determined using flow cytometry and normalized to human neutrophils that did not receive CHIPS. Data are mean±s.d. Significance was calculated using ANOVA, and when needed, followed by Kruskal–Wallis post-test for multiple comparison and displayed as *P<0.05, ****P<0.0001 and NS (not significant).

To assess the *in vivo* therapeutic potency of CHIPS, the immune complex-mediated Arthus reaction model (Köhl and Gessner, 1999; Bestebroer et al., 2010b) was used in hC5aR1^KI^ I mice. The resulting inflammatory response and neutrophil recruitment in the Arthus reaction is mainly C5a mediated. By simultaneously administering ovalbumin (OVA) intravenously (i.v.) and rabbit anti-OVA IgG intraperitoneally (i.p.), an immune complex-mediated type 3 hypersensitivity reaction is induced that leads to the activation of the complement system and the generation of C5a (Köhl and Gessner, 1999; Bestebroer et al., 2010a). An Arthus reaction was successfully induced in hC5aR1^KI^ mice as reflected by the influx of neutrophils to the peritoneal cavity (Fig. 2A). Administration of CHIPS reduced the number of neutrophils recovered from the peritoneal cavity of hC5aR1^KI^ mice (Fig. 2A). Some mice that received CHIPS showed suboptimal inhibition of neutrophil migration, whereas a single mouse showed no evident decrease in neutrophils recovered compared to untreated mice (Fig. 2A).

As *S. aureus* also colonizes rabbits (McCarthy and Lindsay, 2013), it is possible that the rabbit anti-OVA IgG fraction used to induce the formation of immune complexes also contains specific antibodies against CHIPS with potentially neutralizing capacities. To this end, we determined the presence of anti-CHIPS antibodies in the rabbit anti-OVA IgG used. Although the rabbit IgG fraction did contain very low levels of anti-CHIPS antibodies (Fig. 2B), the presence of these anti-CHIPS antibodies only slightly neutralized CHIPS *in vitro* and evidently did not neutralize CHIPS *in vivo* (Fig. 2A,C). Taken together, our investigations demonstrate the therapeutic potential of CHIPS by inhibiting C5a-mediated neutrophil migration *in vivo* in hC5aR1^KI^ mice after inducing an Arthus reaction. A subsequent preclinical study would be necessary to determine the safety of administering CHIPS as a therapeutic agent before moving to a phase I trial.

### CHIPS in preclinical models and human volunteers

To assess the safety of CHIPS, preclinical safety experiments were conducted in non-human subjects, before administration in humans. In all of the animal toxicology studies, we did not observe any CHIPS-related toxicologically significant changes in clinical observations, body weight, food consumption, hematology, coagulation, blood chemistry parameters, ophthalmoscopy, electrocardiograms, macroscopic or microscopic pathology or behaviour (a full preclinical assessment is disclosed in the supplementary Materials and Methods). Notably, a transient decrease in mean arterial blood pressure (40%) was observed in beagles receiving a high dose of 20 mg/kg^-1^ CHIPS (supplementary Materials and Methods). However, mean arterial blood pressure returned to normal within 5 min post-dosing. Overall, these results suggest that side effects induced by CHIPS are unlikely to be observed in human subjects. Consequently, the safety of CHIPS was subsequently studied in a set of six human subjects during a phase I clinical study.

**Fig. 3.**
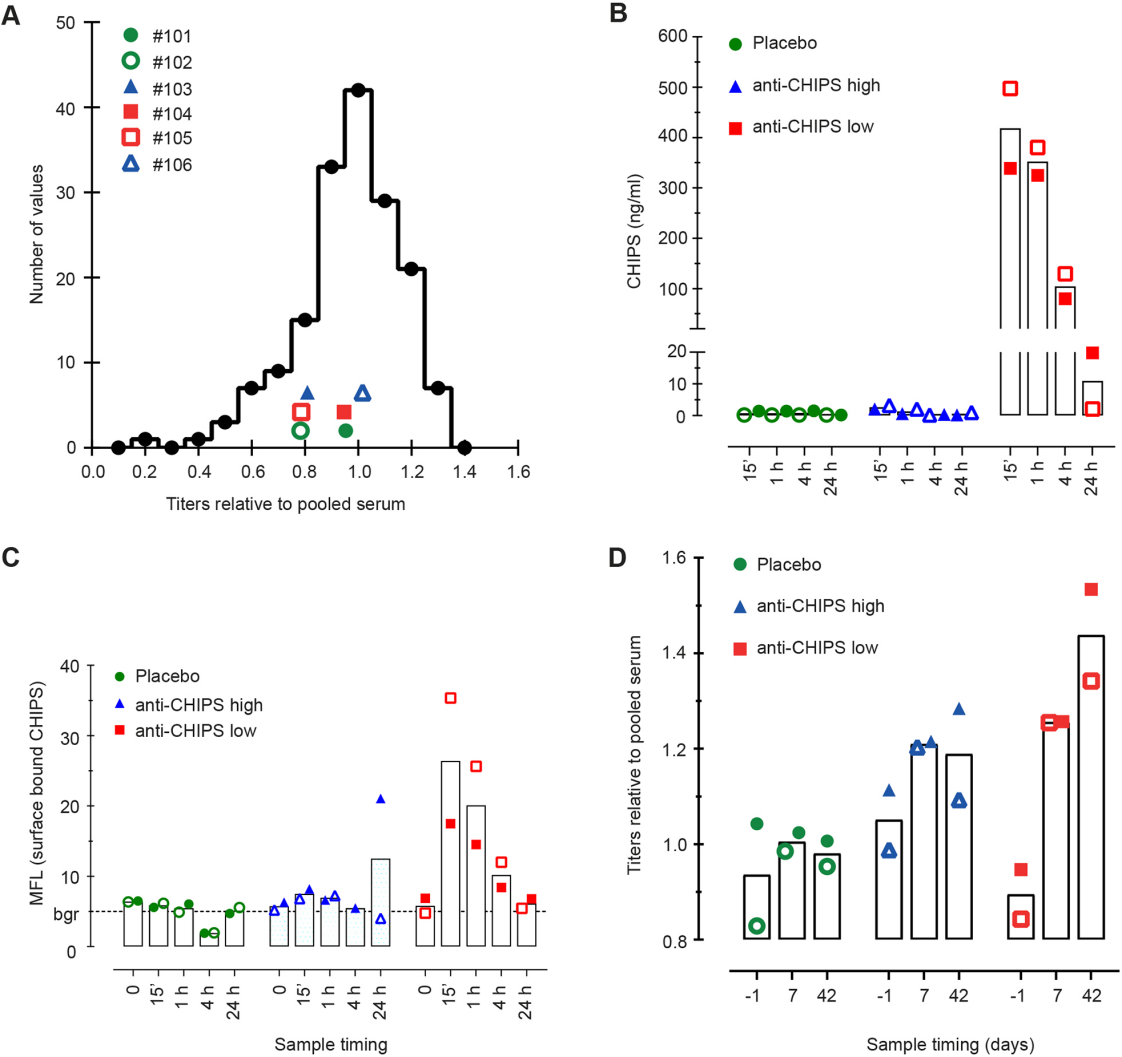
**CHIPS and anti-CHIPS antibodies in humans.** (A) Frequency distribution of IgG anti-CHIPS titer in healthy human donors (*n*=168). The titer was defined as the log dilution that gives an absorbance of OD 0.300 after the subtraction of background value. Titers were depicted relative to the mean human pooled serum (HPS) titer (3.75). The anti-CHIPS antibody titer of the six subjects before study entry are depicted in the same graph for comparison. The ▪ represents subjects that had low anti-CHIPS antibodies (anti-CHIPS low), ▴ represents subjects with high anti-CHIPS antibodies (anti-CHIPS high) and the ● represents subjects in the placebo group. Open and closed symbols differentiate between receivers in each group. (B) Pharmacodynamics of CHIPS detected in the sera of the volunteers. CHIPS was measured by a specific capture ELISA at various time points after intravenous injection of CHIPS. (C) CHIPS is recovered on the surface of peripheral blood neutrophils. At various time points after intravenous injection, the presence of CHIPS bound to the surface of neutrophils was detected with rabbit-anti-CHIPS antibodies. Values are expressed as mean fluorescence (MFL) of gated neutrophils in EDTA whole-blood samples. The background MFL value for the secondary FITC-labeled conjugate was 6. (D) Immunogenicity of CHIPS in healthy human subjects. Specific IgG titers towards CHIPS were determined in all subjects before trial start, 7 and 42 days after close of trial and are depicted relative to HPS.

*S. aureus* is commonly present as a commensal bacterium in humans and the *chp* gene is present in the majority of *S. aureus* strains (de Haas et al., 2004; van Wamel et al., 2006). As a result, most, if not all humans, carry pre-existing anti-CHIPS antibodies (Wright et al., 2007; den Reijer et al., 2013; Verkaik et al., 2010a). However, the anti-CHIPS antibodies present in human sera have been shown to interfere with CHIPS function *in vitro* (Wright et al., 2007). As a consequence, the presence of anti-CHIPS antibodies could neutralize CHIPS or induce an antibody-mediated immune reaction *in vivo*, hampering CHIPS function. To better understand how subject titers relate to the general population, anti-CHIPS IgG titers were determined in sera collected from 168 human volunteers. As expected, anti-CHIPS IgG was detected in all 168 healthy volunteers, and the data resembled a Gaussian distribution (Wright et al., 2007) (Fig. 3A). To limit undesired effects *in vivo*, only subjects with low anti-CHIPS titers were included in the phase I study. The definition of a low antibody titer was set at 3.92 or less, as part of the exclusion criteria, and was based on the average anti-CHIPS titers determined in pooled human serum (*n*=10), defined as the log of the serum dilution that gives an absorbance value of 0.300 in the ELISA. To this end, we determined anti-CHIPS antibody titers in study subjects before they received CHIPS (Fig. 3A). Accordingly, anti-CHIPS IgG titers from subjects were within the normal range of tested sera and were representative of the anti-CHIPS IgG titers of the general population (Fig. 3A). The anti-CHIPS antibody titers in subjects were considered low enough to not affect the safety assessment of CHIPS.

We initiated a phase I randomised double-blind placebo-controlled clinical study in a limited number of volunteers. Based on the toxicology studies, the administration of a single low dose of 0.1 mg/kg^−1^ CHIPS was considered safe and administered in a cohort of six subjects, of whom two received a placebo and four received CHIPS. First, we determined the presence of CHIPS in sera of the volunteers during different time points post-CHIPS administration. In only two out of the four subjects that received the CHIPS protein (subjects 104 and 105) could CHIPS be detected 15 min post-i.v. injection, with a gradual decline after 1 h (Fig. 3B). CHIPS was not detected in the sera of subjects 103 and 106 (Fig. 3B). These observed differences in the detection of CHIPS in the blood of the subjects seem to correlate with their initial level of anti-CHIPS antibodies. We hypothesized that the higher anti-CHIPS antibody titers hamper the detection of CHIPS by ELISA. It is possible that the epitope recognized by either the capture monoclonal or the detecting polyclonal anti-CHIPS antibody is occupied by anti-CHIPS antibodies of the subjects. For analysis and explanatory purposes, from now on we will divided the four volunteers into two separate groups based on their anti-CHIPS antibody titer; anti-CHIPS low (subjects 104 and 105) and anti-CHIPS High (subjects 103 and 106). The measured CHIPS serum concentration in subjects 104 and 105 were also potentially an underestimation due to the interference of pre-existing anti-CHIPS antibodies. In addition, for subjects 104 and 105, which had detectable levels of CHIPS 15 min post i.v. injection, CHIPS concentrations dropped a 2-log fold over the course of 24 h (Fig. 3B). These data show that CHIPS is taken up systemically within 15 min and cleared 24 h post i.v. administration. We calculated a predicted half-life of CHIPS to be at least 1.5 h in humans.

CHIPS binds the C5AR1 on human neutrophils with high affinity *ex vivo* (Postma et al., 2004). In addition, CHIPS binds the formyl peptide receptor 1 (FPR1) present on neutrophils. However, the CHIPS recognition motif is different for both receptors and normal surface expression of C5AR1 is much higher compared to FPR1 (de Haas et al., 2004; Postma et al., 2004; Haas et al., 2004). The binding of CHIPS to its target could be hampered by circulating antibodies *in vivo*. In order to assess whether CHIPS interacts with its cellular targets, we determined the binding of CHIPS *in vivo* on neutrophils of the subjects. The presence of CHIPS on the surface of neutrophils was determined at various time points post-CHIPS administration using a rabbit anti-CHIPS antibody (Haas et al., 2004). Notably, the binding of CHIPS on the surface of neutrophils was only detected in subjects with a low anti-CHIPS antibody titer (subjects 104 and 105) (Fig. 3C). It is possible that the circulating anti-CHIPS antibodies present in serum also interfere with the direct detection by the specific anti-CHIPS antibody or even the direct association with the C5AR1 on neutrophils. Therefore, the lack of a direct detection cannot exclude the absence or presence of CHIPS bound to the receptors in the individuals with high anti-CHIPS antibody titers. Overall, we show that CHIPS binds circulating human blood neutrophils, confirming the interaction with target cells *in vivo*.

All tested subjects had pre-existing anti-CHIPS antibodies. As a specific antibody response is mediated against CHIPS, it is likely that a re-challenge with CHIPS will lead to an increase in antibody titers. To determine the immunogenicity of CHIPS, anti-CHIPS serum titers were measured during different time points pre- and post-CHIPS administration. An increase in anti-CHIPS titer was observed in individuals receiving CHIPS that had a naturally low anti-CHIPS antibody titer (subjects 104 and 105) pre-CHIPS administration (Fig. 3D). The rapid boost of circulating IgG titers by the staphylococcal protein CHIPS in humans indicates high immunogenicity and pre-existing memory, supporting a concept of expected exposure to secreted staphylococcal proteins starting at an early age (den Reijer et al., 2013; Verkaik et al., 2010a; Verkaik et al., 2010b).

### CHIPS induced adverse effects in humans

**Fig. 4.**
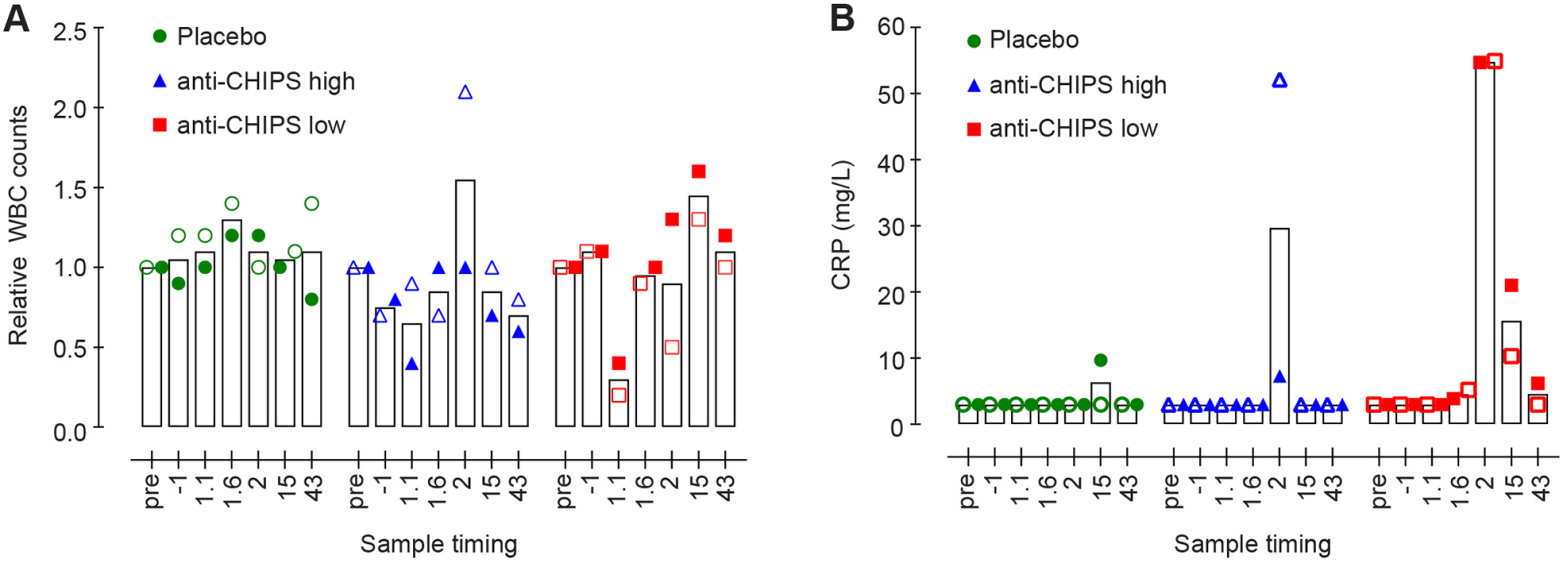
**CHIPS possibly induces leukocytopenia and increased CRP levels in humans.** (A,B) Levels of circulating peripheral WBCs (A) and serum inflammation marker CRP (B). At various time points after intravenous injection of CHIPS, WBC counts and CRP measurements were performed (1.1 and 1.6 indicate 1 day and 1 h or 1 day and 6 h, respectively). The data for WBCs are expressed relative to the value at T=0 and data for CRP are expressed in mg/ml. The ▪ represents subjects that had low anti-CHIPS antibodies (anti-CHIPS low), ▴ represents subjects with high anti-CHIPS antibodies (anti-CHIPS high) and the ● represents subjects in the placebo group. Open and closed symbols differentiate between receivers in each group.

The administration of CHIPS in human subjects was tolerated by two subjects (subjects 103 and 104), moderately tolerated in subject 105 but subject 106 (subject with a high anti-CHIPS antibody titer) developed serious symptoms, directly after the CHIPS infusion, that were diagnosed as an anaphylactic reaction (see University Medical Center Utrecht Department of Medical Microbiology protocol JPD-003/002/NL). No adverse events were reported in subjects receiving the placebo. To determine whether the subjects developed a CHIPS-mediated inflammatory response, white blood cell (WBC) count and C-reactive protein concentration (CRP) were measured pre- and post-dosing. CHIPS induced a moderate transient leukocytopenia in the subjects receiving CHIPS that resolved within 6 h (Fig. 4A). Within the group of subjects that received CHIPS there was a mild increase in CRP (average of 42 mg/ml^−1^) at day 2 post CHIPS dose compared to controls. CRP levels returned to normal when subjects were screened during follow up at day 15 (Fig. 4B). This indicates that there was indeed an inflammatory response upon CHIPS administration.

### Circulating immune complexes and increased serum tryptase

**Fig. 5.**
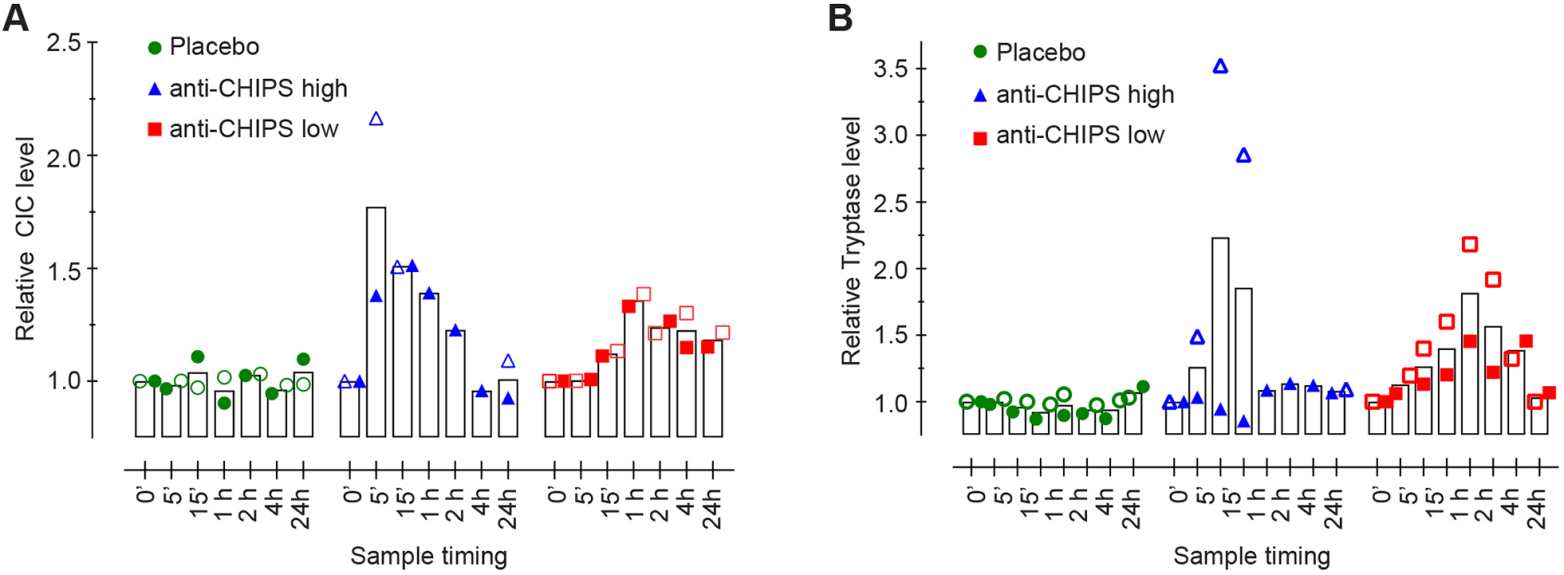
**Adverse effects of CHIPS as measured by levels of CICs, and mast cell marker tryptase.** (A,B) At various time points after intravenous injection of CHIPS, specific assays were performed for CICs (A) and mast cell marker tryptase (B). The ▪ represents subjects that had low anti-CHIPS antibodies (anti-CHIPS low), ▴ represents subjects with high anti-CHIPS antibodies (anti-CHIPS high) and the ● represents subjects in the placebo group. Open and closed symbols differentiate between receivers in each group.

Mast cells play a central role in anaphylaxis and other allergic conditions. Immune complexes can activate mast cells by Fc receptor (FcR) crosslinking and through the activation of complement and the generation of C5a (Jancar and Sanchez Crespo, 2005). Circulating immune complexes (CICs) induce the abundant secretion of the serine proteinase tryptase by mast cells, which can be used as an indicator of anaphylaxis. As all subjects had pre-existing anti-CHIPS antibodies, we evaluated whether intravenous administration of CHIPS leads to the formation of CIC. Circulating immune complexes were detected in the subjects receiving intravenous CHIPS (Fig. 5A). Subject 106, who suffered an anaphylactic reaction following the administration of CHIPS, showed the highest CIC levels, contrary to subjects 104 and 105 who remained at baseline. CICs were also detected in subject 103, who had the highest anti-CHIPS antibody titer but reported only minor adverse effects. No CICs were detected in subjects that received the placebo.

Subsequently, we measured the serum tryptase levels in the subjects. An increase in serum tryptase concentration was detected in all subjects receiving CHIPS except subject 103, whose levels reached a maximum at ∼10 min post-dose and continued to drop to baseline levels after 24 h (Fig. 5B). Notably, subject 106 had the highest levels of tryptase, which correlates with the high levels of CICs measured. These data suggest that CHIPS administration in subjects with high circulating anti-CHIPS titers results in an inflammatory response and adverse effects; however, a high anti-CHIPS titer is not predictive for adverse effects as exemplified by subject 103. Owing to these effects, the study was stopped and no further administrations of CHIPS were undertaken.

## DISCUSSION

The involvement of C5a and C5AR in different disease processes has been described previously (Guo and Ward, 2005). Directly or indirectly blocking the generation of C5a, or directly blocking C5AR1, might serve as interventions in diseases in which abnormal C5AR1 stimulations play an important role. A well-described humanized monoclonal antibody against the complement protein C5 is currently used as a treatment for paroxysmal nocturnal hemoglobinuria (Hillmen et al., 2006; Wijnsma et al., 2019). This monoclonal antibody, also known as eculizumab, binds and prevents the activation of C5, thereby interfering with the upstream generation of C5a. However, a potential downside of adopting eculizumab is that it completely blocks the terminal complement pathway by obstructing the cleavage of C5. As a result, patients receiving eculizumab are 1000-fold to 2000-fold more susceptible to invasive meningococcal disease (McNamara et al., 2017). Internalization and subsequent degradation within FcR-expressing cells also pose a challenge when using eculizumab or other antibodies as forms of treatments (Wijnsma et al., 2019). Depending on the type of C5a- or C5AR1-mediated disease, prolonged inhibition of the complement system might not be desirable as eculizumab has a half-life of 93 h (Wijnsma et al., 2019). Other monoclonal antibodies targeting C5a have also been described and have been suggested to have therapeutic potential (Colley et al., 2018). Furthermore, the C5AR1 antagonistic peptide CCX168 was tested in clinical trials and shown to be well tolerated with promising results (Jayne et al., 2017; Bekker et al., 2016). Therefore, other non-antibody means of targeting C5a or C5AR can be of interest as therapeutic agents and should be further investigated.

In this study, we describe a divergent approach and hypothesized that we can directly inhibit C5AR by using a bacterial secreted virulence factor. Unfortunately, our findings suggest that the staphylococcal-secreted CHIPS is not suitable as a model virulence factor-based therapeutic agent for systemic use in humans. This is probably because of the presence of relatively high levels of pre-existing circulating CHIPS antibodies in humans, which resulted in the development of a hypersensitivity reaction. Leukocytopenia, increased CRP and increased tryptase levels have been observed after the administration of CHIPS. Therefore, this phase I trial had to be stopped and our initial aim to test with higher CHIPS doses was aborted. As a result, we can only present individual data and observations from four subjects. Our observations and analysis of the data suggest antibody titer-dependent adverse effects. Thereby, we continued and classified the four subjects in two groups based on anti-CHIPS low or anti-CHIPS high titers.

A previous study has shown that anti-CHIPS IgG titers were one of highest out of a total of 19 tested staphylococcal proteins (Verkaik et al., 2009). In addition, they showed that there is no difference in anti-CHIPS antibody titers in sera between non-carriers or persistent *S. aureus* carriers from healthy people (Verkaik et al., 2009). Therefore, the adverse effects are probably not related to the subject's *S. aureus* carriage status at the time of the phase I trial. However, we did not check for *S. aureus* carriage during our phase I trial. The relatively high quantity of anti-CHIPS antibodies are likely to be important, as is the difference in quality of these anti-CHIPS antibodies with respect to the potential interference in neutralizing the functionality of CHIPS as a C5aR1-binding protein that prevents C5a-mediated cell activation (Wright et al., 2007).

The circulating CHIPS antibody-mediated adverse effects are an unfortunate drawback of using a bacterial protein such as CHIPS, as compared to, for example, monoclonal antibodies. Despite the neutralizing effect of anti-CHIPS antibodies, we were able to detect significant serum concentrations of the CHIPS. Using a phage-library technique we could identify seven main hotspot regions within the CHIPS that are recognized by human antibodies (Gustafsson et al., 2009). This led to the development of a CHIPS variant that has a 180-fold decreased IgG titer while retaining the biological functionality of blocking C5AR1 signalling and inhibiting C5a-induced chemotaxis (Gustafsson et al., 2010). However, despite developing a version of CHIPS that has low interaction with pre-existing anti-CHIPS antibodies, the high immunogenicity of CHIPS could probably limit its suitability for therapies requiring a single administration.

The development of a human C5aR1^KI^ mouse made it possible to assess the suitability of CHIPS as a therapeutic agent in C5AR1-mediated diseases. However, human C5aR1^KI^ mice could also be used to assess CHIPS as a virulence factor and better understand the contribution of CHIPS to staphylococcal pathophysiology. The use of our human C5aR1^KI^ mouse has already contributed to our understanding of staphylococcal pathophysiology by elucidating the *in vivo* role of the human C5AR1-interacting staphylococcal bi-component toxin HlgCB (Tromp et al., 2018). Besides CHIPS, other staphylococcal proteins have been suggested as potential therapeutic agents for a variety of inflammatory diseases. Previous studies demonstrated that staphylococcal proteins that intervene in C5 complement activation (SSL7 and Ecb) or the FcγR (FLIPr-like), also proved to be effective inhibitors in a murine Arthus model (Bestebroer et al., 2010b; Stemerding et al., 2013; Jongerius et al., 2007). However, as pre-existing circulating antibodies against many, if not all, staphylococcal immune evasion proteins are present in all humans (van Wamel et al., 2006), the use of these staphylococcal proteins as therapeutic agents is probably severely hampered. However, staphylokinase from staphylococci, and streptokinase from streptococci, are two bacterial proteins with thrombolytic activity and are used in the clinic. Human subjects possess low levels of circulating and neutralizing antibodies against staphylokinase, and do mount an immune response (Declerck et al., 1994). In addition, antibodies and neutralizing activity against bacterial virulence factors can last up to 6 months post-administration, as exemplified by streptokinase (Mainet et al., 1998). Therefore, even if the primary dose is tolerated, re-administration should be avoided. Despite the drawbacks of using staphylococcal immune evasion molecules, other bacterial virulence factors have been shown to be possibly applicable as therapeutics. The *S. pyogenes* virulence factor Immunoglobulin G-degrading Enzyme of *S. pyogenes* (IdeS) ablates the humoral immunity by cleaving and inactivating IgG (von Pawel-Rammingen et al., 2002). Even though humans carry anti-IdeS antibodies, IdeS treatment also effectively neutralizes IdeS-specific IgG (Winstedt et al., 2015). IdeS was suggested as a way of helping to prevent antibody-mediated injury to allografts. However, during the combined phase I and II trials, a total of 38 serious adverse effects in 15 patients were witnessed (Jordan et al., 2017). The use of IdeS consistently reduced or eliminated donor-specific antibodies to desirable levels, allowing transplantation from a human leukocyte antigen-incompatible donor (Jordan et al., 2017). Although bacterial immune evasion molecules are not suited for direct use as therapeutic compounds, future molecules based on the bacterial anti-inflammatory proteins could very well be potential new candidates. Knowledge of the exact mechanisms of action and the active sites can lead to the development of small molecule anti-inflammatory drugs based on bacterial virulence factors.

## MATERIALS AND METHODS

### Ethics statement

The randomized controlled trial study protocol (JPD-003/002/NL) and amendments were approved by an independent ethics committee. The study was performed in compliance with the ‘Declaration of Helsinki’ (Scotland, October 2000) and OECD Principles of Good Laboratory Practice, and applicable regulatory regulations. For neutrophil isolation, approval was obtained from the medical ethics committee of the University Medical Center Utrecht (METC-protocol 07-125/C, approved March 01, 2010; Utrecht, The Netherlands). The use of animals was approved by the National Ethical Committee for Animal Experiments and performed according to the guidelines of the Central Animal Facility of Utrecht University (Project AVD115002016565).

### Isolation of rabbit anti-OVA IgG

IgG was purified from rabbit anti-chicken-egg albumin delipidized whole antiserum (Sigma-Aldrich, C6534) using multiple runs over a 1 ml Protein-A HiTrap column (GE Healthcare Life Sciences) on an ÄKTA fast protein liquid chromatography system (GE Healthcare Life Sciences). Rabbit IgG was eluted from the column with 0.1 M citric acid (pH 3.0) and collected fractions were neutralized with 1 M Tris-HCl, pooled and dialyzed against PBS. Protein concentration was determined at 280 nm using a molar extinction coefficient of 1.35 for rabbit IgG.

### Peritoneal Arthus reaction and neutrophil migration

Human C5aR1KI mice were generated and characterized as described previously (Tromp et al., 2018). The Arthus reaction was initiated upon i.v. injection in hC5aR1^KI^ mice (male and female) of 100 μl of OVA (20 mg/kg^−1^ of body weight; Sigma-Aldrich) immediately followed by an i.p. injection of 800 μg of undiluted rabbit anti-OVA IgG (Sigma-Aldrich, C6534-2ML) in 500 µl PBS. For mice in the CHIPS group, 60 μg CHIPS was administered i.p. 30 min before the initiation of the Arthus reaction and simultaneously with OVA i.v. For the control group, PBS was administered i.v. and i.p. Mice were euthanized by CO_2_ suffocation 6 h after the onset of the peritoneal Arthus reaction and the peritoneal cavity was washed two times with 5 ml ice-cold Roswell Park Memorial Institute medium (RPMI) with 0.1% human serum albumin (HSA; Albuman 200 g/l-1, Sanquin)/5 mM EDTA. Peritoneal fluid was recovered and centrifuged at 260 ***g*** for 10 min to collect the exudate cells. Cell pellets were resuspended in 500 µl buffer and counted with trypan blue in a TC20 automated cell counter (Bio-Rad). Cells were stained, in the presence of an 1:30 Fcγ-receptor blocker, with anti-mouse CD45-APC (clone 30-F11, 1:200, BD Biosciences; 559864), anti-mouse Gr1-PE (1A8, 1:125, BD Biosciences; 551461), anti-mouse F4/80 FITC (BM8, 1:33, eBioscience; 11-4801-82), 1:15 anti-human C5aR-FITC (clone S5/1, Bio-Rad; MCA1283F), isotype rat-IgG2a-FITC (1:20, R&D Systems; IC006F) and rat-IgG2b-PE (1:20, BD Biosciences; 553989). Samples were analyzed by flow cytometry. Collected peritoneal cells were washed with PBS and the cell number adjusted to 5×10^6^ cell/ml^−1^. Cytospin slides were prepared with 50 μl 5×10^4^ cell suspension and stained with Diff-Quick. The percentage of neutrophils was determined by flow cytometry analysis and confirmed by the number of neutrophils based on morphology following Diff-Quick staining. Mouse neutrophils were isolated from bone marrow as described previously (Spaan et al., 2013b, 2014). Briefly, bone marrow cells were collected by flushing the femurs and tibias with 10 ml of ice-cold Hank's balanced salt solution+15 mM EDTA+30 mM HEPES+0.1% HSA. A two-layer Percoll density gradient (2 ml each in PBS) composed of 81% and 62.5% was used to enrich neutrophils from the total leucocyte population. Interphase between 62.5% and 81% was collected. Cells were washed once with buffer and resuspended in RPMI1640 with 0.1% HSA. Staining of bone marrow cells was performed as described above.

### Preclinical assessment of CHIPS toxicity in animal models

Conventional preclinical toxicology studies were performed to investigate the safety of intravenous CHIPS. These included: (1) a study examining the effects of CHIPS on various cardiovascular and respiratory parameters in one group of three anesthetized beagle dogs – the dogs were administered CHIPS in incremental doses of 0.2, 2.0 and 20 mg/kg^−1^, infused intravenously over 1 min at ∼30-min intervals; (2) a behavioral (‘Irwin’) test in mice in which CHIPS was administered as a single intravenous injection to male ICR CD-1 mice (three per group) at doses of 7.5, 25 and 75 mg/kg^−1^ in order to assess effects on general behavior [an additional group received an equivalent volume (10 ml/kg^−1^) of vehicle (0.9% w/v sterile saline)]; (3) an acute intravenous toxicity study in rats in which 96.1 mg/kg^−1^ CHIPS was i.v. administered as a single dose (the maximum practically achievable due to volume considerations) to five male and five female rats; (4) an acute intravenous toxicity study in mice in which 96.1 mg/kg^−1^ CHIPS was i.v. administered as a single dose to five male and five female mice; (5) a 7-day intravenous bolus preliminary toxicity study in rats (24 males and 24 females, maximum dose 10 mg/kg^−1^); (6) a 7-day intravenous bolus toxicity study in rats (76 males and 76 females, maximum dose 10 mg/kg^−1^); (7) a 7-day intravenous bolus dose range-finding study in dogs (two males and two females, maximum dose 20 mg/kg^−1^); and (8) a 7-day intravenous bolus toxicity study in dogs (12 males and 12 females, maximum dose 20 mg/kg^−1^).

### Inclusion of human volunteers

A full description of the study population, including the number of subjects and inclusion, exclusion and removal criteria, is outlined in the University Medical Center Utrecht Department of Medical Microbiology protocol JPD-003/002/NL. Briefly, inclusion criteria for healthy volunteers were as follows: (1) adult males within an (2) age range of 18 to 50 and (3) a body mass index of 18-30/kgm^−2^. Medical screening was divided into two parts. Subjects were screened for anti-CHIPS antibody titers. Only subjects with a low titer (equal or lower to 3.92, defined as the log of the serum dilution that gives an absorbance value of 0.300 in the ELISA) were screened for the second part within 3 weeks before dosing and this screening included data obtained from medical histories, a physical examination, measurements of blood pressure, heart rate, respiration and temperature, an alcohol breath test, blood and urine tests, an electrocardiogram (ECG) and drug screening.

### Admission and follow up

A full description of the admission and follow up, treatments and stopping rules are described in the University Medical Center Utrecht Department of Medical Microbiology protocol JPD-003/002/NL. Briefly, six selected subjects (four receiving CHIPS and two controls) were admitted to the Clinical Pharmacology Unit (Kendle, Utrecht, The Netherlands) on the day before dosing. Baseline measurements, including those from blood samples (for safety), urinalysis, interim medical histories, physical examinations, vital signs and ECGs were obtained. On the day of dosing, CHIPS (0.1 mg/kg^−1^ administered as a single dose of sterile frozen isotonic saline solution containing CHIPS at a concentration of 5 mg/ml^−1^) or placebo (0.9% NaCl) was administered by intravenous infusions over 5 min. Subjects were connected to a telemetry system for cardiac monitoring from 30 min before dosing until 4 h after the start of dosing. The blood pressure of subjects was measured continuously using a Finapres from 5 min before dosing until 30 min after the start of dosing. Vital signs were measured and ECGs were obtained at certain time points during the admission period. For safety, clinical status and laboratory values (haematology, biochemistry, coagulation and urinalysis) of all subjects were monitored. Adverse events were documented and characterized according to their severity and relationship to CHIPS or placebo. The subjects were discharged at 24 h after dosing. Two weeks after dosing, subjects returned to the Unit to evaluate vital signs, ECGs, blood and urine and anti-CHIPS antibody levels. A follow up visit was scheduled 6 weeks after dosing.

### Cloning and expression of CHIPS

CHIPS was cloned and expressed as described previously (de Haas et al., 2004; Haas et al., 2004). Briefly, the CHIPS gene (chp; GenBank: AF285146.1), without the signal sequence, was cloned into the pRSET vector directly downstream of the enterokinase cleavage site and before the EcoRI restriction site by overlap extension PCR. Bacteria were lysed with CelLytic B Bacterial Cell Lysis/Extraction Reagent (Sigma-Aldrich) and lysozyme according to the manufacturer's instructions. The histidine-tagged protein was purified using a nickel column (HiTrap Chelating HP, 5 ml, Amersham Biosciences) following the manufacturer's instructions and then cleaved with enterokinase (Invitrogen). Samples were checked for purity and the presence of protein using 15% SDS-PAGE (Mini-PROTEAN 3 System, Bio-Rad) and InstantBlue ISB1L (Merck) staining.

### Purification of CHIPS for intravenous use

Full-length CHIPS was expressed in *E. coli* containing the coding sequence of CHIPS directly downstream of the PelB coding sequence in a growth medium consisting of soya peptone and yeast extract in 8 l fermentation media. CHIPS was isolated both from the growth medium and the cells by a two-stage cation-exchange purification process followed by a desalting step. The bacterial cell pellet was resuspended in phosphate buffer [30 mM (pH 7.0)], containing NaCl (10 mM), dithiothreitol (10 mM), and then frozen. This was subsequently thawed at 37°C, incubated on ice and sonicated. After centrifugation at 26,000 ***g***, an amber-colored ‘cell’ supernatant was recovered. The supernatant was diluted fourfold with 30 mM phosphate buffer and passed over a Source S-30 column. The material was eluted with a phosphate buffer salt gradient, and fractions containing CHIPS were combined and purified further by using a polishing column with a shallow salt gradient. Fractions containing CHIPS with a purity greater than 97% [as ascertained by high-performance liquid chromatography (HPLC)] were combined and passed through a Sephadex G-25 desalting column to remove phosphate and any excess of sodium chloride. Endotoxin was removed by gentle shaking over resin (Bio-Rad) and the preparation was sterilized through ultra-filtration. We confirmed the purity of the fractions using HPLC-mass spectrometry on a μbondapac CN-RP column with a mobile gradient phase consisting of water-TFA to Methanol-TFA. The end product was diluted with sterile saline to the desired concentration and stored at −20°C.

### Isolation of human blood

Polymorphonuclear leukocyte blood obtained from healthy volunteers was collected into tubes containing sodium heparin (Greiner Bio-One) as anticoagulant. Heparinized blood was diluted 1/1 (v/v) with PBS and layered onto a gradient of 10 ml Ficoll (Amersham Biosciences) and 12 ml Histopaque (density 1.119 g/ml^−1^; Sigma-Aldrich). After centrifugation (320 ***g*** for 20 min at 22°C), the neutrophils were collected from the Histopaque phase and washed with ice-cold RPMI 1640 medium containing 25 mM HEPES buffer, L-glutamine (Invitrogen) and 0.05% HSA. The remaining erythrocytes were lysed for 30 s with ice-cold water, after which 10×PBS was added to restore isotonicity. After washing, cells were counted and resuspended in RPMI-1640/0.05% HSA at 10^7^ neutrophils/ml^−1^.

### Determining circulating immune complexes, C-reactive protein and serum tryptase

CICs were determined by two different ELISAs from Quidel: the CIC-C1q Enzyme Immunoassay is based on the principle that complement fixing ICs will bind to immobilized human C1q-purified protein; and the CIC-Raji Cell Replacement Enzyme Immunoassay measures ICs containing C3 activation fragments by using a monoclonal antibody (mAb) that specifically binds the iC3b, C3dg and C3d activation fragments of C3 in a manner that is analogous to the classical Raji cell CR2-binding reaction. The data from both assays were combined and results were expressed relative to the value at time-point 0. CRP levels were determined by the diagnostic department according to standard protocols. Serum-derived tryptase (both α and β form) was measured on the UniCAP-100 using the ImmunoCA technology (Pharmacia Diagnostics). The normal geometric mean for serum tryptase in healthy controls is 5.6 μg/l^−1^. Results were expressed relative to the value at time-point 0.

### ELISA for anti-CHIPS antibodies and CHIPS levels

Rabbits were immunized with recombinant CHIPS using Freund's Complete Adjuvants and boosted with Freund's Incomplete Adjuvants. Bleedings were checked for reactivity with CHIPS by ELISA as described for human anti-CHIPS antibodies. From the final bleeding, IgG was purified by standard Protein G (Pharmacia) affinity chromatography according to the manufacturer's instructions. For the anti-CHIPS ELISA, microtiter plates (Greiner) were coated with 50 μl CHIPS per well at 1 μg/ml^−1^ in PBS overnight at 4°C. All wash steps were performed three times with PBS/0.05% Tween 20 and subsequent incubations were performed for 1 h at 37°C. Plates were blocked with 4% bovine serum albumin (BSA)/PBS/0.05%Tween 20, washed and incubated with sera or antibodies diluted in 1% BSA/PBS/0.05% Tween 20. Bound antibodies were detected with goat anti-human-IgG-horseradish peroxidase (HRP) conjugated (1:5000, SouthernBiotech; 2040-05) with 3,3′,5,5′-tetramethylbenzidine (TMB) as the substrate. The reaction was stopped with H_2_SO_4_ and the absorbance was measured at 450 nm in a Bio-Rad ELISA reader. For the capture ELISA, microtiter plates were coated with 50 μl α-CHIPS mAb 2G8 at 3 μg/ml^−1^ in PBS overnight at 4°C. Plates were blocked with 4% BSA/PBS/0.05% Tween 20, washed and incubated with diluted samples and a twofold dilution range of CHIPS as standard in 4% BSA/PBS/0.05% Tween 20. Subsequently, plates were incubated with 0.33 μg/ml^−1^ rabbit α-CHIPS IgG and goat anti-rabbit-IgG-HRP conjugated (1:5000, SouthernBiotech; 4030-05). Bound antibodies were quantified with TMB as substrate, the reaction stopped with 1 N H_2_SO_4_ and optical density was measured at 450 nm on a Bio-Rad ELISA reader.

### Statistical analysis

Statistical analyses were performed using Prism 7.0 (GraphPad Software). Flow cytometric analyses were performed using FlowJo (Tree Star Software). Significance was calculated using analysis of variance (ANOVA) followed by Kruskal–Wallis as post-test correction for multiple comparison. All statistical methods with regards to the human trials are described in the University Medical Center Utrecht Department of Medical Microbiology protocol JPD-003/002/NL.

## Supplementary Material

Supplementary information
