# Supplementary material for: Pre-existing antibody-mediated adverse effects prevent the clinical development of a bacterial anti-inflammatory protein
Source: Dis Model Mech. 2020 Sep 28;13(9):dmm045534. doi: 10.1242/dmm.045534 (PMC7541340; doi:10.1242/dmm.045534)
Supplement: Supplementary information [file dmm-13-045534-s1.pdf]

Supplementary Materials and Methods

Supplementary Text 1: Preclinical assessment of CHIPS as a therapeutic agent

CHIPS successfully dampened the C5AR-dependent Arthus reaction in a mouse model expressing the hC5AR1. To allow testing of CHIPS in human volunteers, preclinical safety experiments in non-human subjects were required. In none of the toxicology animal studies did administration of CHIPS cause any CHIPS related toxicologically significant changes in clinical observations, body weight, food consumption, hematology, coagulation, blood chemistry parameters, ophthalmoscopy, electrocardiograms, macroscopic or microscopic pathology or behavior. The effects of CHIPS on various cardiovascular and respiratory parameters in anesthetized beagle dogs was examined. In the dogs receiving low dose CHIPS (0.02 and 2 mg/kg-1) there was no evidence of cardiovascular or respiratory effects when compared to infusion of vehicle (isotonic saline). Following intravenous administration of 20 mg/kg-1 CHIPS a transient decrease in mean arterial blood pressure (40%) was recorded approximately ~1 min after start of administration. Mean arterial blood pressure levels returned to pre-dose levels within ~5 min following the start of dosing. The effect on blood pressure coincided with transient, inconsistent changes in heart rate. One dog was administered a repeat intravenous dose of CHIPS (20 mg/kg-1) ~30 min following the first administration of CHIPS. Transient effects on cardiorespiratory parameters similar to those recorded following the first dose were not apparent after the repeat administration of CHIPS. However, the second administration produced a prolonged reduction in mean arterial blood pressure, reaching a maximum of 18% at ~30 min following the second administration. In this animal only, 12 min following the repeated administration of CHIPS a generalized skin reaction appeared consistent with some form of mild allergic reaction. The results of this study suggested that cardiorespiratory effects are unlikely to be observed in the human subjects in the used dose range (0.1 mg/kg-1). Furthermore, any effects that might occur were expected to be transient and reversible.

Disease Models & Mechanisms • Supplementary information
